# Transformer networks enable fast and robust dictionary generation for multiparametric cardiac mapping with variable timing

**DOI:** 10.1016/j.jocmr.2026.102757

**Published:** 2026-06-08

**Authors:** Pauline Calarnou, Amaury George, Costa Georgantas, Angela Rocca, Gabriel Paffi, Augustin C. Ogier, Roger Hullin, Jonas Richiardi, Ruud B. van Heeswijk

**Affiliations:** a Department of Radiology, Lausanne University Hospital (CHUV) and University of Lausanne (UNIL), Lausanne, Switzerland; b Cardiology Service, Cardiovascular Department, Lausanne University Hospital (CHUV) and University of Lausanne (UNIL), Lausanne, Switzerland

**Keywords:** deep-learning, transformer, Quantitative MRI, cardiac, multiparametric mapping, acceleration, in-line reconstruction

## Abstract

**Background:**

Cardiac multiparametric mapping often relies on dictionary matching. When the acquisition timing varies due to electrocardiographic or navigator gating, dictionaries must be simulated for each acquisition, which is time-consuming and limits their in-line clinical utility. Deep learning can be used to accelerate this dictionary generation. In this work, we implement and evaluate a transformer network that leverages self-attention to capture long-range dependencies and compare its performance against a previously proposed fully connected multi-layer perceptron (FC-MLP).

**Methods:**

Transformer (6 layers, 700,674 trainable parameters) and FC-MLP (3 hidden layers, 300–300–75 units) networks were implemented, optimized, and trained on synthetic timing intervals generated through extended phase graph (EPG) simulation. Network performance was quantified as mean average percent error (MAPE) versus EPG simulations in silico, as a correlation in a mapping phantom and through Bland-Atman analysis in 138 patients (age 67±13 y, 50 F) at 3T. Inference time was recorded. Finally, the full mapping pipeline was integrated directly on the MR (magnetic resonance) scanner.

**Results:**

EPG dictionary simulations required 30±2 minutes per subject, whereas the transformer completed inference in 13.2±0.4 s on CPU and 89.2±0.4 ms on GPU, representing a >100-fold acceleration. In the phantom experiments, the transformer achieved near-ideal correlation with the EPG dictionary (slope 1.00 vs 1.02 for FC-MLP) and demonstrated superior accuracy in Bland-Altman analysis, with smaller bias (1.8 ms vs 13.6 ms) and narrower limits of agreement. Across patient datasets, the transformer exhibited tighter error distributions for T_1_ and T_2_ (median MAPE [IQR]: 0.37%[0.09%–0.76%] and 0.50%[0.25%–0.91%] compared to FC-MLP (2.04%[1.61%–2.55%] and 3.03%[2.40%–3.92%], p<3×10⁻¹⁷² for both), respectively. Correlation between navigator skips and MAPE was also lower for the transformer, indicating greater robustness. In vivo transformer-dictionary maps were visually indistinguishable from EPG-dictionary maps. Bland-Altman analysis in the myocardium confirmed very small biases for the transformer-dictionary maps (-1.70ms for T_1_, 0.23 ms for T_2_) versus substantially larger errors for FC-MLP-dictionary maps (18.41 ms for T_1_, 1.16 ms for T_2_).

**Conclusions:**

The transformer network significantly accelerates cardiac multiparametric map reconstruction while maintaining accuracy comparable to EPG simulations and superior to the FC‑MLP. Its robustness to variable acquisition timing and minimal bias support clinical feasibility, and successful on‑scanner integration demonstrates the potential for routine in‑line deployment of quantitative T_1_-T_2_ mapping.

## Graphical abstract

**Figure:**
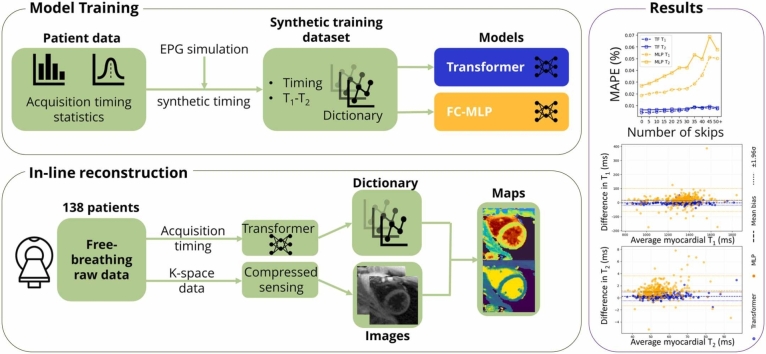

## 1 Introduction

Multiparametric mapping of the heart enables simultaneous estimation of multiple relaxation times (such as T_1_ and T_2_), accelerating tissue characterization and removing cross-confounders. In dictionary-based multiparametric mapping (such as magnetic resonance (MR) fingerprinting) [1], all possible combinations of the relaxation times are simulated as a dictionary, and measured signals in each pixel are matched to the closest dictionary entry.

PARMANav (PArametric Radial Mapping with Navigator gating) [2] implements this approach by acquiring 25 navigator-gated single-shot golden-angle radial source images with inversion recovery (inversion time=68ms for the subsequent image), T_2_ preparation (TE=23/45/70 ms), or no preparation modules. Due to navigator rejection and electrocardiogram (ECG) trigger detection failure, there are often multiple heartbeat “skips” between the acquisitions of the source images that significantly vary the sequence timing and thus the evolution of the magnetization. Because of these effects, as well as regular heart rate variability, extended phase graph (EPG) [3] simulations are used to generate a scan-specific T_1_–T_2_ dictionary. While accurate, this process takes ∼30 min per map. To accelerate this, we trained an encoder-decoder transformer, [4] optimized it with simulated patient-based timing, and evaluated it against a fully connected multi-layer perceptron (FC-MLP) on 138 patient datasets (52,193,120 entries).

## 2 Methods

### 2.1 Neural network architecture

**Fig. 1:**
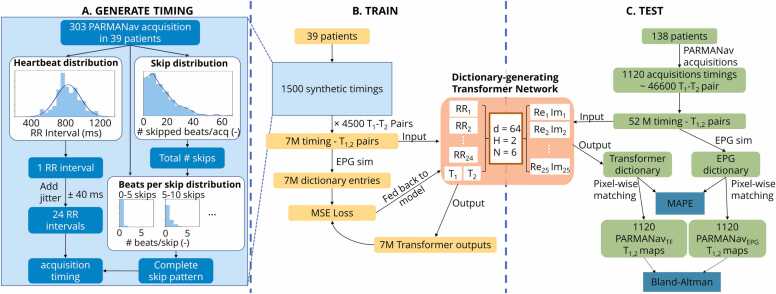
Overview of the data preparation, training and testing of the transformer model. A) PARMANav timing data from 39 patients were used to characterize the distribution of heartbeat durations and the number of ECG trigger skips (due to navigator rejection or undetected ECG R waves). These distributions were used to generate 7 M synthetic timing pairs. B) EPG simulations of these 7 M pairs were performed and the transformer network was trained on these individual T_1_–T_2_ pairs. C) To test the network, T_1_–T_2_ dictionaries for 138 patients were EPG simulated and predicted with the network. The entire dictionaries were compared with MAPE, while the T_1_ and T_2_ maps were compared through Bland-Altman analysis. *d* embedding dimension, *H* heads, *EPG* extended phase graph, *MAPE* mean absolute percentage error, *MSE* mean squared error, *N* encoder and decoder layers, *PARMANav* PArametric Radial Mapping with Navigator gating

Since PARMANav produces 25 source images, the acquisition-specific T_1_-T_2_ dictionaries must account for the 24 sampling intervals between these source images. We evaluated two architectures for predicting EPG-simulated signals from these sampling intervals and relaxation parameters (Fig. 1). The proposed model was an encoder-decoder transformer (embedding dimension d=64, heads H=2, 6 encoder and 6 decoder layers) with sinusoidal positional encodings. The baseline model was an FC-MLP with three hidden layers (300–300–75 units), rectified linear unit (ReLU) activations, and layer normalization, following the model described by Hamilton et al. [5] Both models take 26-element array inputs (i.e., 24 sampling intervals + a T_1_-T_2_ pair) and output a 50-element signal array (25 complex samples split into real and imaginary components).

We used a mean squared error loss function:

(1) $$L=\frac{1}{50}\sum_{i=1}^{50}{\left({\hat{y}}_{i}-y_{i}\right)}^{2}$$

with dictionary estimate and true $\hat{y},y\in \;R^{50}$. The trained network and source code are available at https://gitlab.com/chuv-tmrr/trafo-dict/.

### 2.2 Training data generation

A synthetic training dataset containing 6990,000 EPG-simulated dictionary entries was generated in Matlab (Mathworks, Natick, Massachusetts) to ensure broad coverage of all possible sequence timings without directly using patient data timings. To generate 1500 acquisitions timings, 303 PARMANav datasets from 39 participants acquired on a clinical 3T scanner (PrismaFit, Siemens Healthineers, Forchheim, Germany) were used (Fig. 1A). In this dataset (with 0–70 heartbeat skips and heartbeat duration 417–1203 ms, see Supplementary Material) we characterized the distribution of the heartbeat durations and skipped ECG triggers (due to navigator rejection or ECG detection failure), using empirical histograms. For the latter, we characterized both the number of skips per acquisition N_skip_ and the number of consecutive heartbeats missed per skip F_skip_. A training timing was then generated by sampling a single heartbeat duration for the 24 intervals from the histogram, varying these with zero-mean Gaussian noise (σ=40 ms), and using the skip distributions to multiply N_skip_ randomly selected intervals with their own skip factor F_skip_.

Each resulting synthetic acquisition timing was paired with 4660 randomly selected T_1_–T_2_ pairs (simple random sampling from 46601 pairs), yielding 6990,000 million synthetic input timings. Finally, we simulated the corresponding MR signals with EPG simulation for each input timing.

### 2.3 Network training

Optuna [6] was used to jointly optimize model hyperparameters for both architectures. The Adam optimizer [7] was used for both models with early stopping (see Supplemental Material for more details).

The validation and test sets were composed of real participant acquisition data. A total of 1120 PARMANav acquisition were collected from 138 participants on the same 3T scanner, with typically eight acquisitions per patient (four different cardiac slices acquired in native and post-gadolinium-based contrast agent (GBCA) injection). The cohort consisted of 72 heart failure patients [8] (age 71±13 y, 27 F, including both heart failure with preserved ejection fraction and with reduced ejection fraction), 27 healthy volunteers (age 67±14 y, 15 F); and 39 heart transplant recipients (age 57±12 y, 8 F). For the validation set, 50,000 dictionary entries were randomly sampled from the transplant recipient group, while the remaining data formed the test set (9320,200 entries). This patient population enabled evaluation across cardiac conditions and acquisition scenarios.

### 2.4 Model evaluation

In the International Society for Magnetic Resonance in Medicine/National Institute for Standards and Technology (ISMRM/NIST) phantom, [9] we compared relaxation times obtained with both deep learning models to the original EPG-generated values as well as reference standard inversion-recovery spin-echo (for T_1_) and spin-echo (for T_2_) values under both constant heartbeat and random skip conditions.

Inference speed was recorded for both models, and the simulation time for the EPG-based dictionary was recorded. To ensure fair comparison, all dictionaries were generated using CPU (central processing unit) only.

Model performance in predicting patient dictionaries was further evaluated using two complementary metrics. The mean absolute percentage error (MAPE) of an entire dictionary was calculated for each parameter $j\in \left\{1,2\right\}$ corresponding to T_1_ and T_2_ as:

(2) $${\mathrm{MAPE}}_{j}=\frac{100}{N}\sum_{i=1}^{N}\varepsilon_{i,j};\varepsilon_{i,j}=\;\frac{\left|T_{i,j,\mathrm{EPG}}\;-\;T_{i,j,\mathrm{MODEL}}\right|}{T_{i,j,\mathrm{EPG}}}$$

where $\varepsilon_{i,j}$ is the per-parameter relative error defined for each dictionary entry i=1, …, N (N = 46,601) each parameter $j\in \left\{1,2\right\}$, $T_{i,j,\mathrm{EPG}}$ is the input relaxation parameter, and $T_{i,j,\mathrm{MODEL}}$ is the relaxation time obtained when matching the model signal to the closest EPG dictionary entry. MAPE is reported as median and interquartile range and significance differences were tested using Wilcoxon signed-rank tests, with effect sizes quantified via paired rank-biserial correlation (r_bs_). Spearman correlations were calculated between MAPE and the total number of skipped heartbeats for both models to assess the relation between skips and error. P-values were adjusted using a Bonferroni correction for multiple comparisons, with p<0.05 considered statistically significant.

To assess in vivo agreement, we segmented the myocardium in 631 maps from 116 patients and performed Bland-Altman analysis of model-derived against EPG-based T_1_-T_2_ values in these segmentations. Separate analyses were performed for native and post-GBCA maps. Maps were blindly reviewed for visible regional differences by an experienced CMR (cardiovascular magnetic resonance) cardiologist (Panagiotis Antiochos, MD, >10 y experience).

### 2.5 On-scanner implementation

The transformer model was implemented directly on a clinical 3T scanner (PrismaFit, Siemens Healthineers) using the FIRE (Framework for Image REconstruction) protocol. After the acquisition, the raw data was automatically sent to a remote server, where the complete map reconstruction pipeline was executed. Image and map reconstruction times were recorded. The resulting maps were automatically transferred back to the scanner for in-line display.

## 3 Results

The EPG simulations took 30±2 min per acquisition. In contrast, the transformer completed inference in 13.2±0.4 s on CPU and 89.2±0.4 ms on GPU (graphics processing unit), while the FC-MLP required 48±3 ms on CPU and 0.18±0.07 ms on GPU.

In the phantom, the transformer T_1_ and T_2_ values showed higher agreement with the EPG-generated values than the FC-MLP values (T_1_ slope of 1.00 vs. 1.02, Supplemental Figure 1). Bland-Altman analysis confirmed its higher accuracy, with a much smaller T_1_ bias (1.8 ms vs 13.6 ms) and narrower T_1_ limits of agreement (−5.0 to 8.6 ms vs. −40.0 to 67.3 ms).

**Fig. 2:**
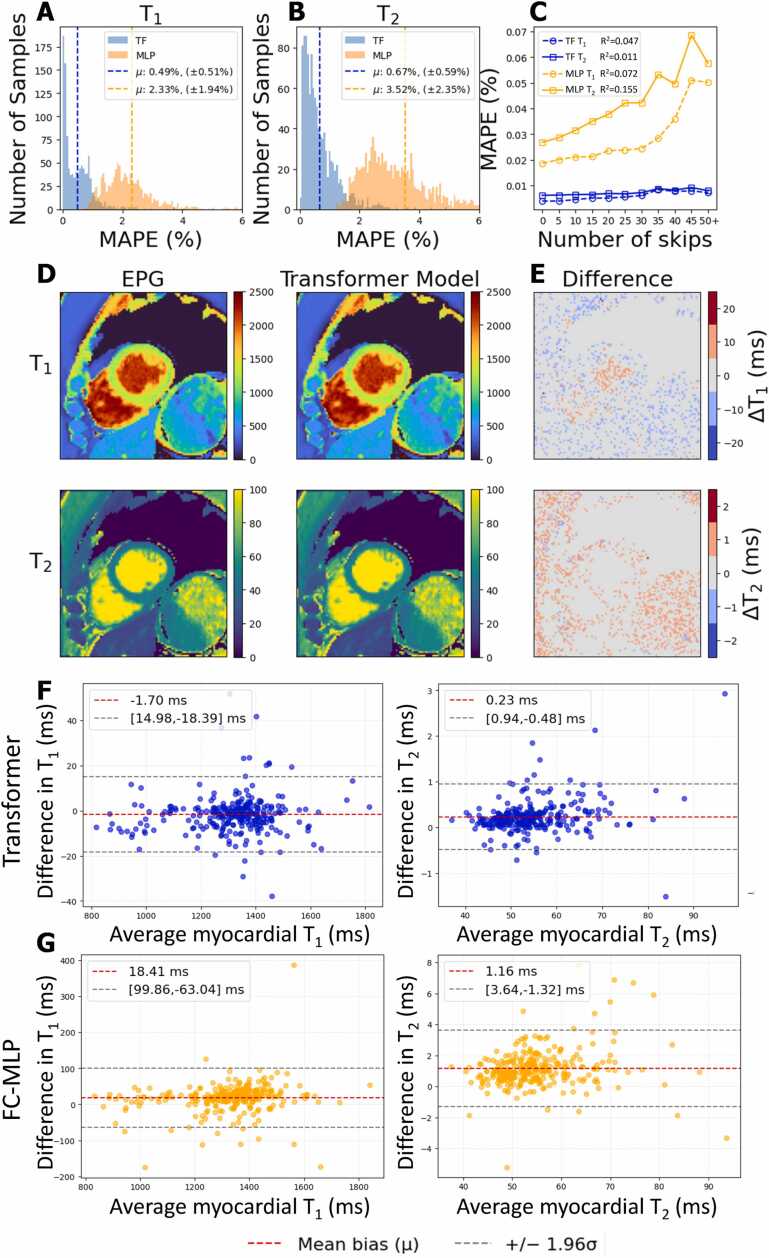
Comparison of the transformer and FC-MLP performance. A-B) Histogram of MAPE across all test datasets, comparing the dictionaries for the transformer (TF) and the FC-MLP (MLP) model to the EPG reference. C) T_1_ and T_2_ MAPE per number of total skips during the scan. R^2^≤0.05 suggests model robustness to skips; residual errors likely stem from geometric conditioning and heart rate variability. D) Sample T_1_ and T_2_ maps created with the PARMANav EPG dictionary and the PARMANav transformer dictionary. The maps are visually nearly identical. E) Difference maps (transformer - EPG) – no systematic regional differences were identified by an experienced CMR cardiologist. F-G) Bland-Altman plots for the myocardial T_1_ and T_2_ values obtained with the transformer and FC-MLP dictionaries compared to the EPG-generated values in native maps. *EPG* extended phase graph, *FC-MLP* fully connected multi-layer perceptron, *MAPE* mean absolute percentage error, *PARMANav* PArametric Radial Mapping with Navigator gating, *TF* transformer

The transformer resulted in tighter T_1_ and T_2_ MAPE limits of agreement, with fewer outliers than the FC-MLP (MAPE_1_=0.37%(0.09–0.76%) and MAPE_2_=0.50%(0.25–0.91) for the transformer and MAPE_1_=2.0%(1.6–2.6%) and MAPE_2_=3.0%(2.4–3.9%) for the FC-MLP, respectively, p<3×10^−172^ and r_bs_>0.85 for both, Fig. 2A). Outlier analysis revealed that larger errors mainly occurred in acquisitions with extensive skipped beats, which were less severe with the transformer than with FC-MLPs. The correlation coefficients between the transformer T_1_–T_2_ MAPE and the total number of navigator skips were 0.22 and 0.10, significantly lower than the FC-MLP coefficients at 0.27 and 0.39 (p<0.006 for all) (Fig. 2B).

The in vivo network-generated and EPG-simulated maps were visually nearly identical upon blinded review (Fig. 2C-E). Most T_1_ and T_2_ differences were 10 ms and 1 ms, respectively, which is the dictionary step size and thus the smallest possible nonzero error.

Myocardial Bland-Altman analysis (Fig. 2F-G) showed that the transformer model yielded very small T_1_ and T_2_ biases (-1.7ms and 0.23 ms, respectively, less than a quarter of a dictionary step) versus substantially larger T_1_ and T_2_ biases with the FC-MLP (18.4 ms for T_1_ and 1.16 ms for T_2_). The FC-MLP had several extreme outliers and larger limits of agreement. The same tendency can be observed in the post-GBCA maps (Supplemental Figure S2). Finally, the in-line implementation was successful and provided maps ∼2 min after the end of the sequence (Supplemental Figure 3). This included compressed sensing reconstruction in Matlab (∼1min30s), dictionary generation using the transformer model (∼10 s) and parametric map computation.

## 4 Discussion

In this proof-of-concept study, the transformer-based model achieved negligible T_1_ and T_2_ bias while accelerating dictionary generation by over 100-fold compared to traditional EPG simulation approach. This reduced end-to-end mapping from ∼30 min to under two minutes (dominated by ∼1m30s of compressed sensing image reconstruction).

The transformer’s superior performance likely stems from its self-attention mechanism, which captures long-range dependencies across non-consecutive RR intervals. Unlike FC-MLPs, which treat each time point independently, the transformer’s multi-head attention learns to weight contributions from any part of the time series, making it robust to varied skip patterns. The FC-MLP architecture was intentionally constrained to replicate the established standard, [5] providing a practically relevant baseline independent of parameter count. The availability of a large synthetic training dataset is also key to the transformer’s performance, as this architecture can struggle in the low-data regime. Outlier analysis suggested a better generalization of the transformer compared to FC-MLP for extreme number of skips.

Clinically, MAPE below 1% is smaller than intrinsic biological variability of myocardial relaxation times, [10] and likely reflects a performance floor established by inherent dictionary discretization, which conflates model error with discretization noise and may mask further architectural improvements. The resulting maps are effectively indistinguishable from the ground truth but available in near real time. This enables clinicians to access T_1_ and T_2_ maps during the same exam session, supporting in-line protocol adjustments and clinical decision-making.

Limitations include the method’s specificity to the PARMANav sequence, with performance on other protocols yet to be validated. Additionally, deriving the training and validation timings solely from single-center transplant recipients represents a minor methodological constraint; future studies should employ stratified validation across diverse populations. This proof-of-concept transformer had a longer inference time than the FC-MLP due to its architectural complexity, but was not optimized for speed and still demonstrates a clinically viable map reconstruction time both on CPU and GPU. However, measured inference times (13.2 s on CPU and 89.2 ms on GPU) establish a performance range that satisfies clinical workflow requirements across modern hardware tiers. In their current implementations, the FC-MLP may suffice when large T_1_–T_2_ changes are expected. While in silico results show timing robustness, phantom validation used a single skip condition; broader characterization is warranted. Finally, while patients with diagnosed arrhythmia were not included in the training data, the broad range of simulated skips beat durations covers the resulting heart rates. Future studies should include specific arrhythmic cohorts to evaluate the model in the presence of large rhythm irregularities.

The current work focused on joint T_1_-T_2_ mapping, and extension to parameters such as B_1_, T_1ρ_, or T_2_* should be feasible. However, this would require exponentially larger dictionaries, potentially presenting computational challenges, which would need to be addressed by reducing the individual training dictionaries.

## 5 Conclusion

We introduced a transformer-based dictionary generator for free-breathing joint T_1_–T_2_ cardiac mapping with PARMANav, achieving high accuracy, excellent agreement with EPG references, and substantial acceleration over conventional EPG simulations. This approach enables robust, clinically viable parametric mapping with end-to-end on-scanner reconstruction in just two minutes.

## Author contributions

**Pauline Calarnou:** Writing – original draft, Methodology, Investigation, Formal analysis, Data curation, Conceptualization. **Amaury George:** Writing – original draft, Methodology, Investigation, Formal analysis, Data curation, Conceptualization. **Costa Georgantas:** Methodology. **Angela Rocca:** Resources. **Gabriel Paffi:** Methodology, Data curation. **Augustin C. Ogier:** Methodology, Data curation. **Roger Hullin:** Resources. **Jonas Richiardi:** Supervision, Methodology, Conceptualization. **Ruud B. van Heeswijk:** Writing – original draft, Supervision, Methodology, Funding acquisition, Conceptualization.

## Ethics approval and consent

Ethics approval was obtained from the Ethics Committee of the Canton of Vaud (CER-VD) of Switzerland under reference numbers 2019–01879 and 2022–00934. All participants provided written informed consent to participate prior to enrollment.

## Declaration of competing interests

The authors declare that they have no known competing financial interests or personal relationships that could have appeared to influence the work reported in this paper.

## Data Availability

The PARMANav images and maps will be made available on Zenodo for the HeartMagic project. [8] The data generation, training, and testing code as well as the trained networks are available on https://gitlab.com/chuv-tmrr/dict-trafo/. Other study data are available on reasonable request from the corresponding author.

## Footnotes

Appendix A Supplementary data associated with this article can be found in the online version at doi:10.1016/j.jocmr.2026.102757.
