# Supplementary material for: Transformer networks enable fast and robust dictionary generation for multiparametric cardiac mapping with variable timing
Source: J Cardiovasc Magn Reson. 2026 Jun 8;28(2):102757. doi: 10.1016/j.jocmr.2026.102757 (PMC13311263; doi:10.1016/j.jocmr.2026.102757)
Supplement: Supplementary file 1 — Supplementary material [file mmc1.docx]

Transformer networks enable fast and robust dictionary generation for multiparametric cardiac mapping with variable timing

Pauline Calarnou^1^, Amaury George^1^, Costa Georgantas^1^, Angela Rocca^2^, Gabriel Paffi^1^, Augustin C. Ogier^1^, Roger Hullin^2^, Jonas Richiardi^1^, Ruud B. van Heeswijk^1^

1. Department of Radiology, Lausanne University Hospital (CHUV) and University of Lausanne (UNIL), Lausanne, Switzerland

2. Cardiology Service, Cardiovascular Department, Lausanne University Hospital (CHUV) and University of Lausanne (UNIL), Lausanne, Switzerland

# Supplementary material

## Training Data Source Characteristics

Synthetic training data were derived from 303 PARMANav datasets (pre- and post-GBCA injection) from 39 heart transplant recipients (age 57±12y, 8F). Median heartbeat durations were 825ms (interquartile range (IQR) 729-958ms, from 417ms to 1203ms), while the heart rate variability (defined as the median of the within-subject standard deviations of regular RR intervals) was 29ms (IQR 16-64ms, from 2ms to 174ms). The median navigator skips per acquisition was 12 (IQR 7-22, from 0 to 70). Importantly, the data from these subjects were not directly used for network training - only the derived timing distributions were used to generate the synthetic training set.

## Synthetic training heart rate pseudo-code

The following algorithm describes the generation of the synthetic acquisition timing patterns used to train the transformer and FC-MLP networks. The process is divided into an offline statistical modelling phase and an online sampling phase.

I. Statistical Modeling of Patient Data

- Baseline Heart Rate: Fit a normal distribution to the mean RR intervals of the clinical transplant cohort to determine the population mean and standard deviation.
- Skip Budget: Fit a Gaussian Kernel Density Estimation (KDE) to the total number of skips per acquisition to model the empirical frequency of skips non-parametrically.
- Skip Patterns: Group the acquisitions into bins based on their total skip count. For each bin, calculate the Empirical Cumulative Distribution Function (CDF) of "beats skipped per interval" to capture the specific rhythm of navigator gating.

II. Synthetic Pattern Generation

- For each desired synthetic sample:

1. Sample baseline_RR from the Normal distribution (clipped to 250–1600 ms).
2. Sample total_skips for the scan from the KDE.
3. For each of the 24 intervals between segments:

- Sample noise from Normal(0, 40 ms) to model intra-scan variability.
- Calculate interval_RR = baseline_RR + noise.
- Sample beats_to_skip from the Empirical CDF matching the total_skips bin.
- Calculate wait_time = (beats_to_skip + 1) * interval_RR.
- Update segment_start_time.

1. Combine the final timing vector with a sampled (T1, T2) pair.
2. Run the Extended Phase Graph (EPG) simulator to generate the target signal label.

## Model Training and Hyperparameter Optimization

Models were trained on 50,000 EPG-simulated samples using the Adam optimizer with a batch size of 1024 on a workstation with a 24-core AMD Ryzen Threadripper PRO 7965WX CPU and an Nvidia RTX 6000 Ada GPU (48 GB GDDR6). The objective function was the mean squared error (MSE) between predicted and ground-truth parameters, with target labels normalized by their L_2_​ norm to improve training stability. An early-stopping mechanism terminated training after 3 (transformer) or 10 (FC-MLP) consecutive epochs without validation loss improvement to prevent overfitting.

Architectures and hyperparameters were determined via 100 trials of Bayesian optimization using the Optuna framework (TPE sampler and MedianPruner). The transformer search space included learning rate and weight decay (10^−5^-10^−3^, log scaling), dropout (0.0-0.5), model dimension_​_ (32-128), layers (2-12), and attention heads (2, 4, 8). This identified the 6-layer architecture (700,674 parameters) as optimal. The FC-MLP was designed to approximate the approach of Hamilton and Seiberlich (2020); its search space included learning rate and weight decay (10^−5^-10^−2^, log scaling) and 3-layer permutations of 75, 150, and 300 units; a 300-300-75 structure was selected.

## Post-hoc Power Calculation

A post-hoc power analysis confirmed that the sample size (n=1120) is sufficient to detect the observed differences between models. The following derivation from the observed effect size (r_bs_​) to statistical power (1−β) was used:

1. Probability of superiority (PS): calculated from the non-parametric effect size (r_bs_​=0.85 for T_1_​; r_bs_​=0.87 for T_2_​) as PS=(r_bs_​+1)/2, yielding PS=0.925 and 0.935, respectively.
2. Cohen’s d estimation: using a Probit transformation (d≈Φ^−1^(PS)×√2​) the effect sizes were d≈2.03 for T_1_ and d≈2.14 for T_2_​.
3. Power calculation: at α=0.05 (two-tailed) with n=1120 and applying the Wilcoxon Asymptotic Relative Efficiency (ARE) correction of 0.955, the statistical power (1−β)>0.999.

To detect a clinically meaningful difference (5%; d=0.50) with 90% power, a sample size of n≈48 would have been sufficient. The current study is therefore substantially overpowered, ensuring that the superior performance of the transformer is statistically robust.

## Figures


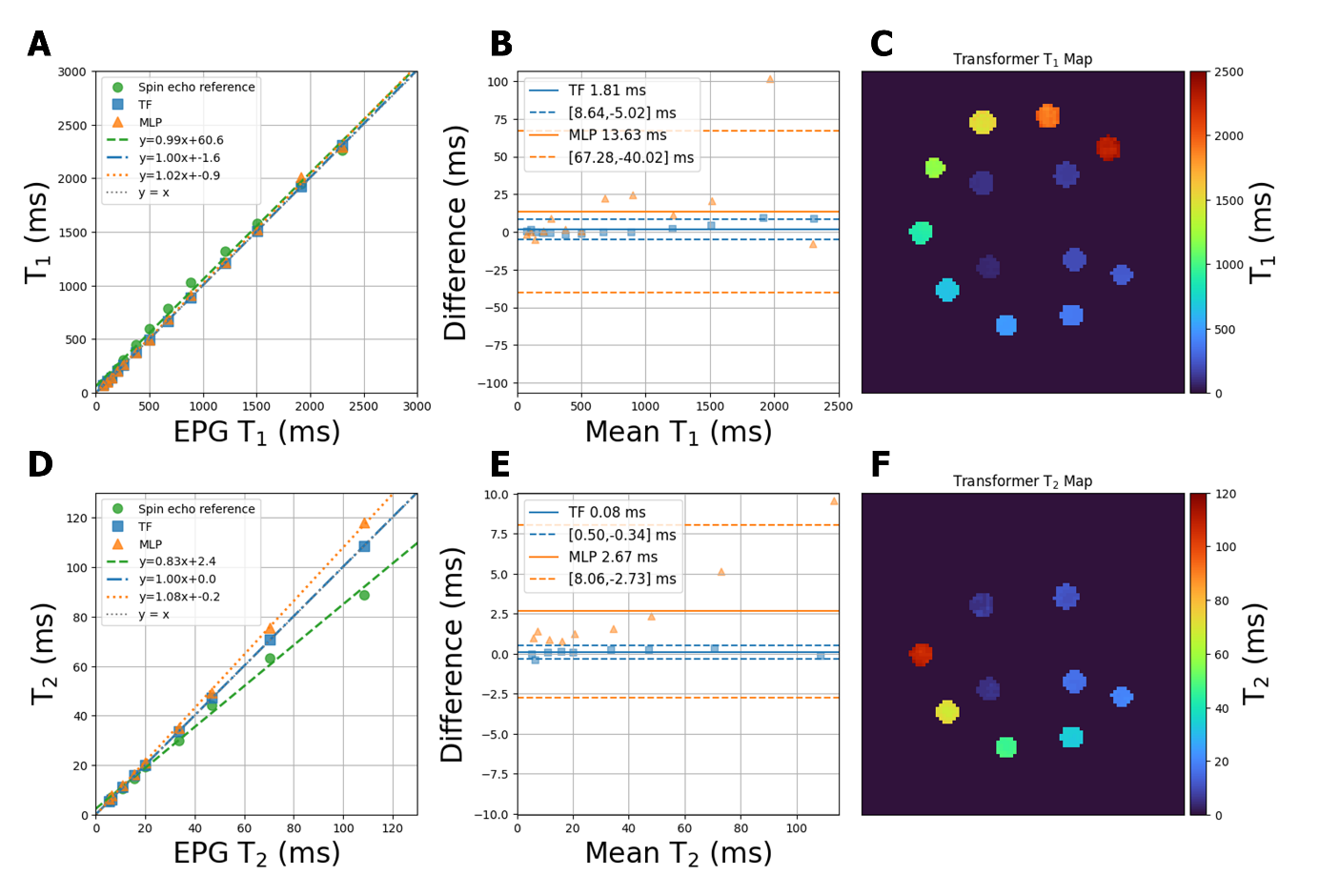


**Figure S1: Agreement of the transformer and FC-MLP model derived maps against the EPG**

**dictionary derived maps in the ISMRM-NIST phantom compared to spin-echo reference values in case of 23 navigator skips. A)** Linear regression of PARMANav T_1_ values obtained with the transformer and the FC-MLP models versus the reference EPG simulation, also compared to gold-standard inversion-recovery spin-echo values. **B)** T_1_ Bland-Altman plot of transformer and FC-MLP versus EPG. The biases (solid lines) and confidence intervals (dashed lines) are reported in the legend. **C)** PARMANav T_1_ map obtained with the transformer dictionary. **D-F)** The same plots as A-C for the T_2_ relaxation time. EPG - extended phase graph; FC-MLP - fully connected multi-layer perceptron; PARMANav - PArametric Radial Mapping with Navigator gating; TF – transformer


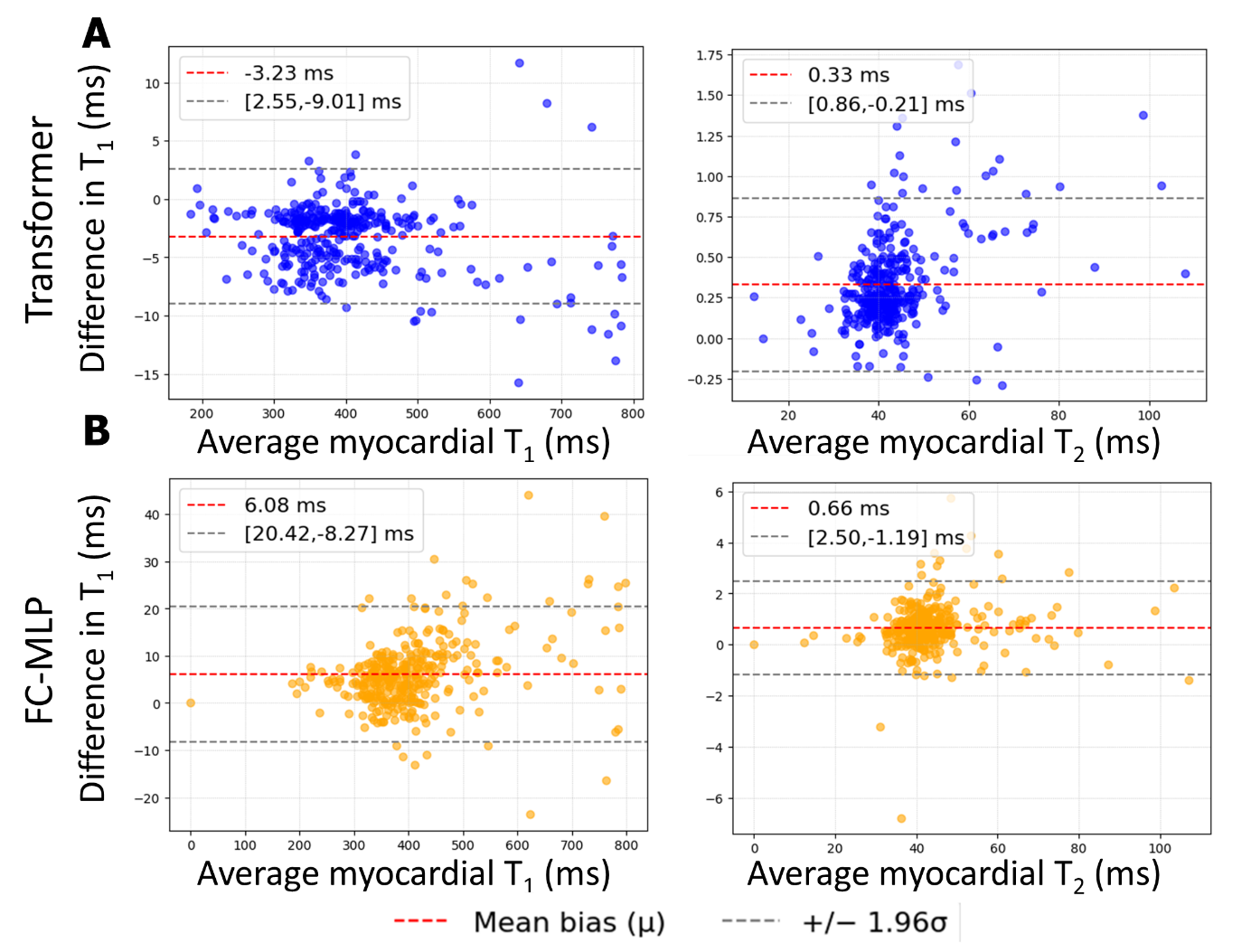


**Figure S2: Bland-Altman plots for the deep-learning dictionary myocardial T_1_ and T_2_ values in post-GBCA.** **A)** T_1_ and T_2_ Bland-Altman plots for the transformer compared to the EPG dictionary. **B)** T_1_ and T_2_ Bland-Altman plots for the FC-MLP compared to the EPG dictionary. Bias and confidence intervals are indicated in the legends. EPG - extended phase graph; FC-MLP - fully connected multi-layer perceptron; GBCA - gadolinium-based contrast agent


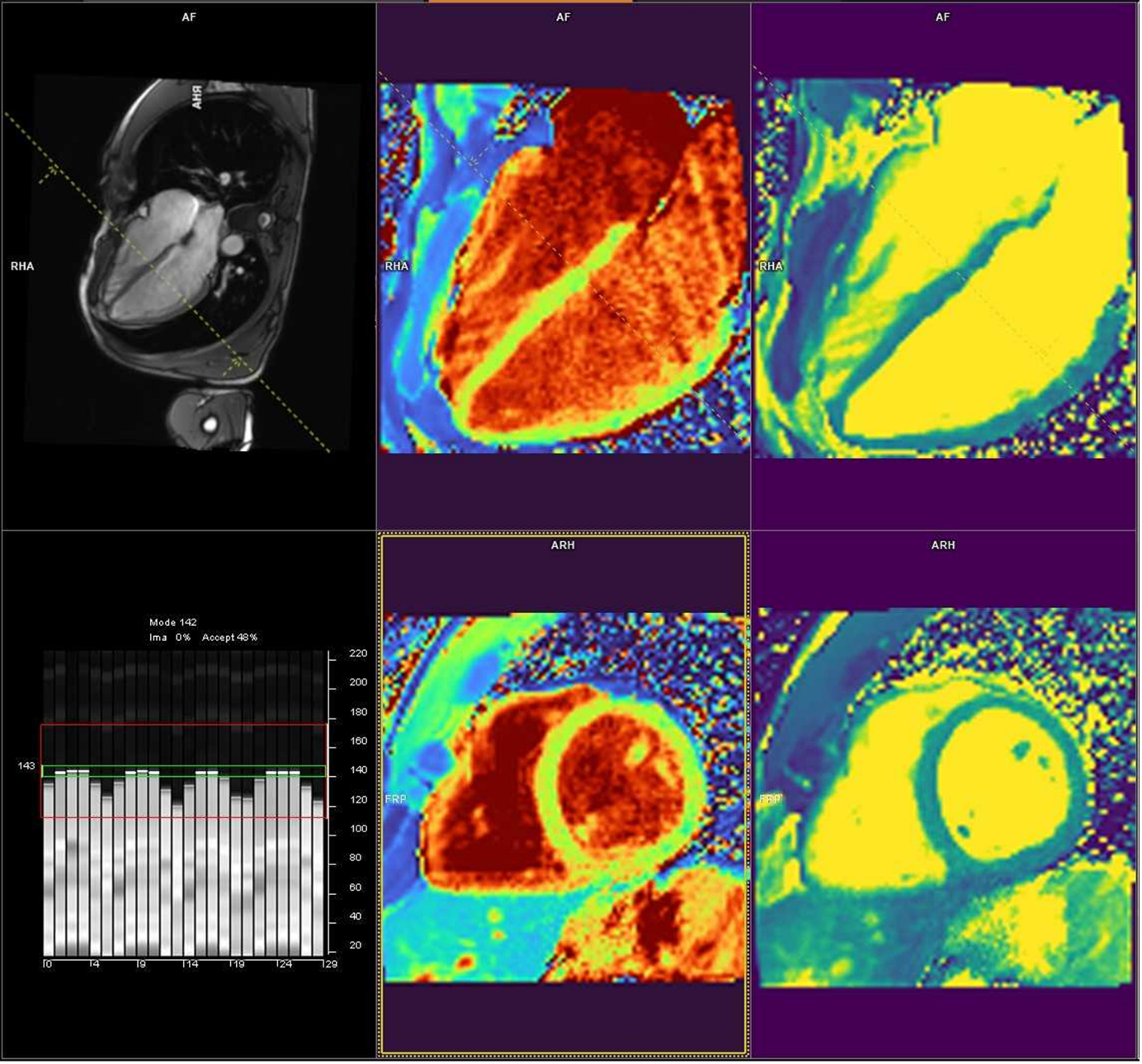


**Figure S3: Screenshot of the reconstructed maps as seen on the MR scanner shortly after the PARMANav maps were acquired in a healthy volunteer**. In the left column a localizer scan and the navigator trace are shown, the middle column shows a four-chamber and mid-level short-axis T_1_ map, and the right column shows the corresponding T_2_ maps. MR – magnetic resonance; PARMANav - PArametric Radial Mapping with Navigator gating
